# A Novel Method for Detecting Duchenne Muscular Dystrophy in Blood Serum of *mdx* Mice

**DOI:** 10.3390/genes13081342

**Published:** 2022-07-27

**Authors:** Nicole M. Ralbovsky, Paromita Dey, Andrew Galfano, Bijan K. Dey, Igor K. Lednev

**Affiliations:** 1 Department of Chemistry, University at Albany, SUNY, 1400 Washington Avenue, Albany, NY 12222, USA; nralbovsky@yahoo.com (N.M.R.); agalfano@albany.edu (A.G.); 2 The RNA Institute, University at Albany, SUNY, 1400 Washington Avenue, Albany, NY 12222, USA; pdey@albany.edu; 3 Department of Biological Sciences, University at Albany, SUNY, 1400 Washington Avenue, Albany, NY 12222, USA

**Keywords:** Raman spectroscopy, chemometrics, early diagnosis, skeletal muscle, Duchenne muscular dystrophy

## Abstract

Duchenne muscular dystrophy (DMD) is the most common form of muscular dystrophy, typically affecting males in infancy. The disease causes progressive weakness and atrophy of skeletal muscles, with approximately 20,000 new cases diagnosed yearly. Currently, methods for diagnosing DMD are invasive, laborious, and unable to make accurate early detections. While there is no cure for DMD, there are limited treatments available for managing symptoms. As such, there is a crucial unmet need to develop a simple and non-invasive method for accurately detecting DMD as early as possible. Raman spectroscopy with chemometric analysis is shown to have the potential to fill this diagnostic need.

## 1. Introduction

Duchenne muscular dystrophy (DMD) is a disease that causes muscles to progressively degenerate and typically affects males. Obtaining a diagnosis for DMD can be complex; involved in the process of diagnosis are family history evaluations and muscle enzyme assays. However, each of these methods faces its own issues. DMD does not always present itself within a family, oftentimes making historical evaluations meaningless. Further, enzymatic assays such as measuring serum creatine kinase (CK) is oftentimes performed for DMD. However, CK is not a specific marker for DMD as this enzyme can be altered in many conditions, including seizures, cardiac, renal, infectious, hormonal, and other neuromuscular disorders [1,2,3,4,5,6]. Alternative testing has been explored, including electromyography which identifies the presence of muscle weakness but not the cause [7] and muscle biopsies which can distinguish between types of muscular dystrophies [8]. However, neither of these tests are definitive, and both are costly as well as invasive for an individual to undergo. It is necessary to diagnose DMD as early as possible due to the progressive nature of the disease, suggesting that the currently available diagnostic methods are inadequate and in need of improvement. A method which is definitive, accurate, non-invasive, and can identify the disease early will be the most beneficial to the patient.

An innovative technique for the simple, accurate, and objective diagnosis of DMD was explored using Raman spectroscopy coupled with chemometrics. The premise of the method was based on the analysis of serum obtained from the *mdx* mouse model compared to serum obtained from similarly aged control mice. This mouse model was chosen due to similar phenotypes experienced compared to humans with DMD. Specifically, within humans, an out-of-frame mutation in the DMD gene results in decreased production of the dystrophin protein in muscle fibers [9,10]; in the *mdx* mouse model, dystrophin is not expressed, thus making the mouse model a sufficient representation commonly used in the literature for exploring and studying DMD without the additional influence of confounding factors which may occur within humans [11,12].

Raman spectroscopy, in combination with chemometric analysis, is used as a novel and accurate method for diagnosing DMD using the well-established *mdx* mouse model. Raman spectroscopy is an exceptional analytical tool first reported in 1928 by C. V. Raman and K. S. Krishnan [13,14] and Grigory Landsberg and Leonid Mandelstam [15]. The combination of Raman spectroscopy and chemometrics offers significant advantages for use as a novel medical diagnostic tool. Raman spectroscopy is well established for capturing the inherent heterogeneity within a sample; this can be accomplished by collecting a large number of spectra across a defined grid on the surface of the sample. This way, one can obtain a spectrum from multiple specific points on the sample. The more spectra that are obtained, the more information is identified. This process allows the user to increase the sensitivity of the method–that is, the overall concentration of a chemical component in the sample may be low, but by probing many small areas of a sample, chances improve for detecting that same component where it is more concentrated in a specific area. Further, this provides the opportunity to identify novel diagnostic biomarkers as well as investigate changes in a specific biological component over time with disease progression. Due to this, the entire composition of a sample can be better studied, thus allowing for the potential to identify components within a sample that could be used for successful diagnostic applications [16].

The utility of Raman spectroscopy for identifying diseases has been widely illustrated [17], including specifically for cancer [18,19], Alzheimer’s disease [16,20,21,22,23], and others [24,25,26,27]. In this work, Raman spectroscopy is combined with a chemometric method called Partial Least Squares Discriminant Analysis (PLS-DA). This method was employed for classifying Raman spectra of serum as either originating from a healthy control mouse or from an *mdx* model mouse. The PLS-DA model is validated using external validation to ensure the model is successful and reaches sufficient levels of diagnostic accuracy. In total, this protocol provides details on how to use Raman spectroscopy and chemometrics to successfully identify DMD in a simple, accurate, and objective manner.

## 2. Experimental Design

**Figure 1:**
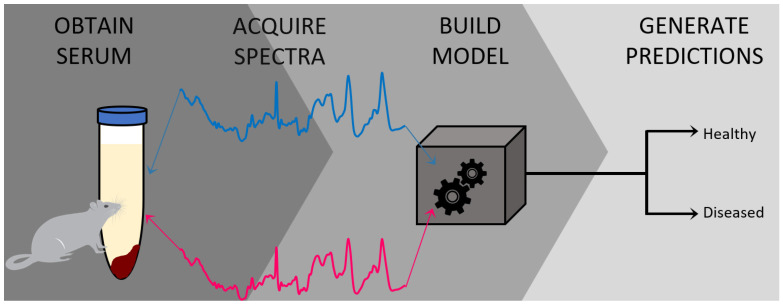
Graphical representation summarizing the protocol described herein, beginning with the collection of blood serum from the mouse donors and subsequent preparation of the serum for collecting Raman spectra. Then, the chemometric model is built and validated to be further used for generating classification predictions.

Herein we describe a method for the accurate and objective diagnosis of DMD using blood serum. The first section of the protocol explains how to isolate and prepare the blood serum samples from wild-type control (C57BL/10ScSnJ) and an *mdx* (C57BL/10ScSn-Dmd<*mdx*>/J) mouse model for analysis. This *mdx* mouse model is the current state-of-the-art preclinical model of DMD that provides milder (early stage) disease condition. Since our goal is to detect the disease at an earlier stage, this model works perfectly to achieve this goal. However, other preclinical models of DMD, including D2.B10 (DBA/2-congenic) DMD *mdx* mouse can be used for this study. The second portion of the protocol describes how to use Raman spectroscopy in combination with chemometric analysis as a successful method for distinguishing control from *mdx* model mice in a simple, accurate, early, and minimally invasive manner. In this protocol example, 14 different mice were used for analysis, with serum obtained and analyzed from each. Of the mice, six were obtained at three months of age, and eight were obtained at 12 months of age. An even number of mice at both ages were either from the *mdx* model of mice or wild-type control. To build the model, Raman spectra from ten mice was used (five control and five *mdx* mice); external validation was performed using Raman spectra obtained from the remaining four mice (two control and two *mdx*). Due to the small number of total samples, data from the different aged mice were evenly distributed across both cross- and external validation. Overall, this protocol describes a method with a strong potential for clinical diagnosis of DMD [28]. An overview of the protocol is illustrated in Figure 1.

### 2.1. Materials and Reagents

- The *mdx* mouse model of Duchenne muscular dystrophy (C57BL/10ScSn-Dmd<*mdx*>/J; The Jackson laboratory, Bar Harbor, ME, USA Stock number 001801) and the counterpart wild type control mouse (C57BL/10ScSnJ; The Jackson laboratory, Stock number 000476).
- 70% Isopropyl alcohol (Fisher Scientific, Waltham, MA, USA, cat. No. A459-500)
- Cotton swabs (Texwipe, Kernersville, NC, USA, cat. no. 18-366-473)
- Dissection tray (EiscoLabs, Victor, NY, USA, cat. no. 2021884)
- Sharp dissecting scissors (VWR, Radnor, PA, USA, cat. no 82027-578-EA)
- Forceps (Fisher Scientific, cat. No. 08-895)
- A 25 gauge needle (BD Syringe, East Rutherford, NJ, USA, cat no. 801015)
- Gilson pipettes (Gilson, Middleton, WI, USA, cat. no. F123601)
- Autoclaved tips (Gilson, cat. no F172310)
- 0.5 mL Eppendorf tubes (Axygen, Union City, CA, USA, cat. no. PCR05B)
- Plain glass microscope slides (Fisher Scientific, cat. no. 12-550-A3)
- Aluminum foil tape (ULINE, Pleasant Prairie, WI, USA, cat. no. S-18880)
- Acetone (Thermo Fisher Scientific, cat. no. A18-500)
- Kimtech science™ Kimwipes™ delicate task wipes (Kimberly-Clark Professional, Roswell, GA, USA, product code 34155)
- Petri dish with clear lid (Fisher Scientific, cat. no. FB0875713)
- Eppendorf™ 0.5–10 μL single-channel pipette (Fisher Scientific, cat. no. 05-412-422)
- Filter tip pipette tips (Fisher Scientific, cat. no. 02-707-473)

### 2.2. Equipment and Software

- EZ Anesthesia AF 9000 Auto flow Anesthesia System (EZ Systems, Palmer, PA, USA, cat. no. EZ-190AF)
- Universal Animal Restrainer (VWR, cat. no. 47750-270)
- Induction chamber for isoflurane inhalation (Vet Equip, Pleasanton, CA, USA, cat. no. 942102)
- Refrigerated tabletop centrifuge (Fisher Scientific, cat. no. 22-029-677)
- Ultra-low temperature freezer (Fisher Scientific, cat. no TSX50086A)
- Renishaw inVia™ confocal Raman microscope (Renishaw, Wotton-under-Edge, England, UK)
- MATLAB^®^ software (MathWorks, Natick, MA, USA)
- PLS_Toolbox software (Eigenvector Research Inc, Manson, WA, USA)
- easyROC web-tool ver. 1.3.1 (http://www.biosoft.hacettepe.edu.tr/easyROC/ (accessed on 6 April 2020)).

## 3. Procedure

### 3.1. Preparation of Blood Serum Samples from Wild Type Control and mdx Mouse Model of DMD

1. Obtain mice from the Jackson Laboratory. In this protocol, 14 mice were used, where six mice were three months old and eight mice were twelve months old.
2. Anesthetize the mice to a surgical plane of anesthesia under isoflurane inhalation using an induction chamber and EZ Anesthesia AF 9000 Auto flow Anesthesia System or following the standard procedure of your IACUC. Verify the depth of anesthesia by establishing the loss of pedal reflex. Then, secure the mouse in a restrainer.
3. Squeeze the skin on the upper thigh of the mice gently but firmly to elevate the Lateral Saphenous Vein while holding the restrainer.
4. Remove the hair using a clipper and swab with 70% alcohol. Next, swab the skin with a small amount of alcohol to help visualize the vein.
5. Locate the lateral saphenous vein as shown [29], puncture the vein with a 25 gauge needle and collect approximately 100 µL of blood from 3-month-old and approximately 150 µL of blood from 12-month-old mice in an Eppendorf collection tube without the use of anticoagulant.
6. Keep the tubes containing the blood samples without any anticoagulant at room temperature in a standing position for 35 min, allowing the blood to clot.
7. Centrifuge the clotted blood samples at 2000 *g* for 15 min in a refrigerated centrifuge.
8. Collect the serum fraction from the supernatant to a fresh tube using a pipette.
9. Aliquot the serum in a small volume and store at −80 °C until analysis, at which point the sample is thawed. The number of freeze/thaw cycles should be minimized as much as possible–it is recommended that a small volume of each donor’s serum is stored in multiple vials rather than the entire volume of the sample stored only in one vial.

### 3.2. Raman Spectroscopic Analysis and Chemometric Detection of DMD

**Figure 2:**
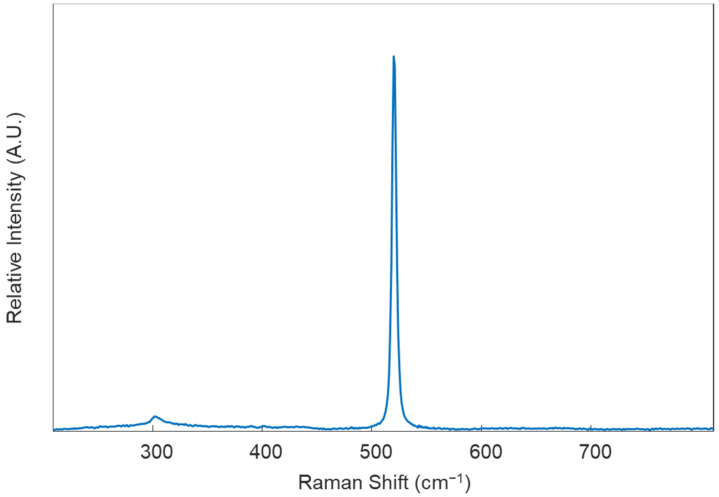
Example Raman spectrum of a silicon standard obtained under excitation using a 785 nm diode laser.

**Figure 3:**
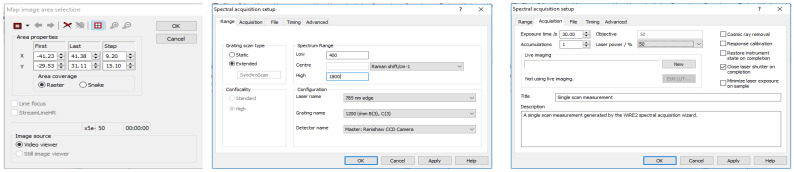
Spectral acquisition parameters for collecting spectra from the blood serum samples.

**Figure 4:**
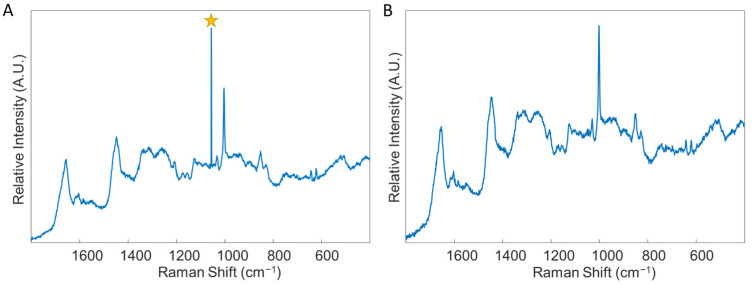
An example of a spectrum with a cosmic ray, denoted by the star (**A**), and an example of a spectrum without any cosmic rays (**B**). Both spectra were obtained through analysis of blood serum. Star: denotes cosmic ray.

**Figure 5:**
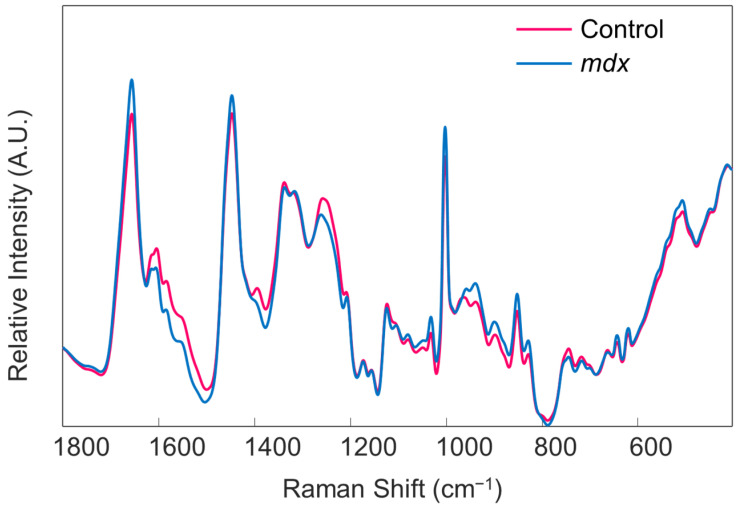
The mean preprocessed Raman spectra of all blood serum samples obtained from the control (pink trace) and *mdx* model mice (blue trace), before mean centering. Reprinted with permission from N. M. Ralbovsky, P. Dey, A. Galfano, B. K. Dey, and I. K. Lednev, Diagnosis of a model of Duchenne muscular dystrophy in blood serum of *mdx* mice using Raman hyperspectroscopy, Scientific Reports, 10, 11734, Copyright (2020) Springer Nature.

**Figure 6:**
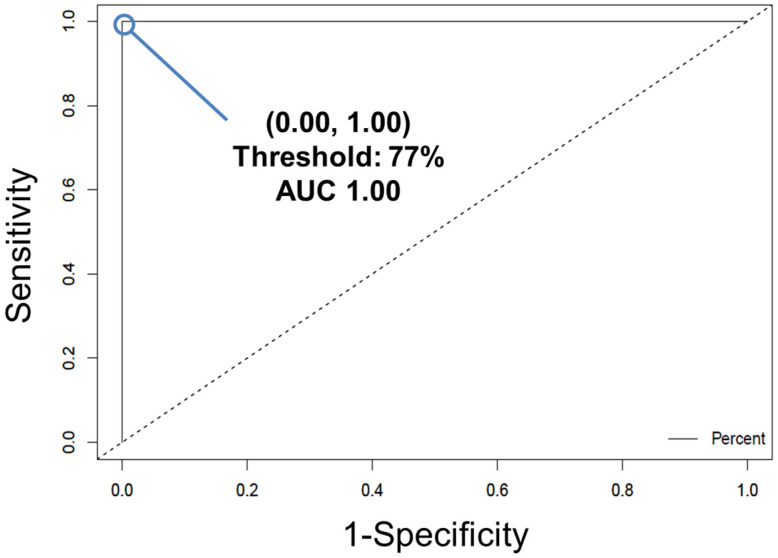
The ROC curve for the cross-validated PLS-DA model, trained to differentiate between diseased and healthy control mice blood serum. The true positive rate (sensitivity) of each potential discrimination threshold is plotted according to each corresponding false positive rate (1–specificity). The optimal threshold is designated by the point at (0.00, 1.00), corresponding to a threshold of 77%. Reprinted with permission from N. M. Ralbovsky, P. Dey, A. Galfano, B. K. Dey, and I. K. Lednev, Diagnosis of a model of Duchenne muscular dystrophy in blood serum of *mdx* mice using Raman hyperspectroscopy, Scientific Reports, 10, 11734, Copyright (2020) Springer Nature.

**Figure 7:**
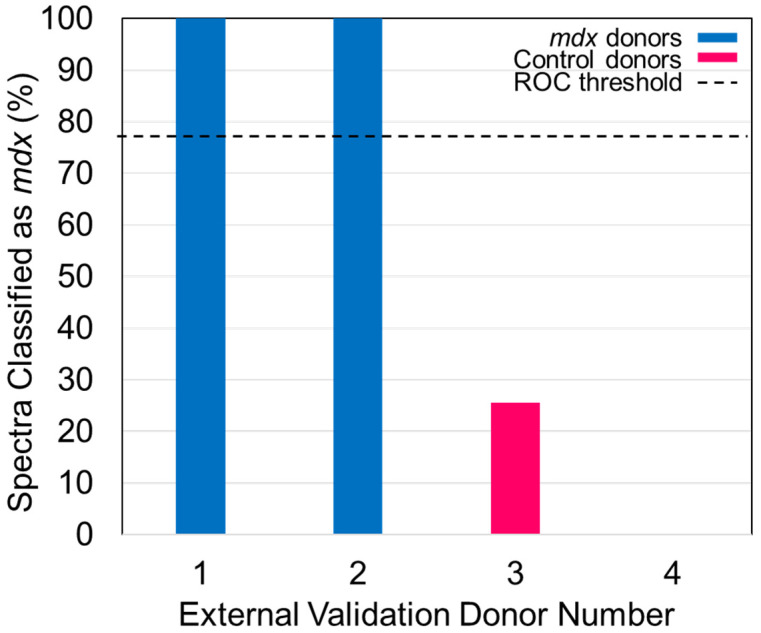
The percentage of spectra classified as belonging to the *mdx* model of mice is plotted as the bar height of each of the donors. The 77% threshold is plotted as the dashed line. Reprinted with permission from N. M. Ralbovsky, P. Dey, A. Galfano, B. K. Dey, and I. K. Lednev, Diagnosis of a model of Duchenne muscular dystrophy in blood serum of *mdx* mice using Raman hyperspectroscopy, Scientific Reports, 10, 11734, Copyright (2020) Springer Nature.

1. Before preparing any samples, the user must divide their donors into two separate groups for analysis: the first group of donors is used to build the classification model and is called the calibration dataset. This group should include roughly 70% of the total number of donors in the study. The second group is used for external validation of the classification model and is thus called the validation dataset. This group encompasses the remaining 30% of the total number of donors. The separation of all donors into the two groups (calibration and validation) should be done randomly.
  *The number of analyzed samples depends on the type of study being performed; for a proof-of-concept study, the number of samples is arbitrary but should minimally exceed five donors per class (healthy, diseased, etc.). For a statistically significant trial, power analysis should be performed to determine the number of donors required.*
  [30]
2. Prepare a microscope slide by covering half of it in aluminum foil tape. Briefly wipe the aluminum foil using acetone and a Kimwipe to remove any debris from the surface; cleaning is highly recommended but not required for a successful experiment. Label the slide with pertinent information, including but not limited to sample type (e.g., serum), donor ID, and date. Place the microscope slide inside the bottom half of a Petri Dish.
3. Deposit between 5 to 10 μL of serum from one donor onto the aluminum foil-covered portion of the microscope slide. It is beneficial to deposit the serum in a thin line rather than a single droplet.
4. Allow the blood serum sample to dry completely overnight within the covered Petri Dish.
  *Samples should be prepared in the same manner for all donors; while differences in sample preparation may not necessarily affect the ability of the method to differentiate between healthy and diseased donors, it is useful to reduce the number of variables in the study to ensure differentiation is based solely on disease state.*
5. Prepare the instrument for analysis. Turn on the laser (set for 785 nm excitation), as well as the instrument, the microscope stage, the microscope light, and the computer to which the instrument is attached. Set the microscope objective to the 50× magnification.
6. Calibrate the instrument using the internal standard (in this protocol, it is a silicon standard). Collect an initial Raman spectrum from the silicon standard using the default collection parameters; there should appear a single peak in the spectrum, around 520 ± 0.5 cm^−1^ (Figure 2). If the single Raman peak is located outside of this range, the instrument will need to be calibrated using the “Quick Calibration” function under the “Tools” tab in Renishaw’s WiRE (Windows-based Raman Environment) software. This software comes with the Renishaw inVia™ confocal Raman microscope and is used here for data collection.
7. Remove the first sample from its Petri Dish and place it on the microscope stage. Next, focus on the sample using the 50× objective.
  *It is best to focus on an area of the sample where the height of the portion of the sample visible under the objective is roughly even. This will allow for better mapping of the sample. In addition, one can determine if the sample height is even by observing which areas of the sample concurrently move in or out of focus when the adjustment knobs are altered.*
8. Conduct spectroscopic mapping of the sample. First, in the WiRE software, select “Measurement”, “New”, and finally “Map Acquisition”. Then, in the “Video” display screen of the sample, highlight an area of the sample that is about 4800 μm^2^ and choose the raster collection feature.
9. Set the map to collect spectra in a roughly 10 × 5 grid to collect a total of 50 spectra from the sample. The area selected and grid size chosen should remain relatively consistent throughout the analysis of all samples in the study.
  *Oftentimes, depending on the probed location of the sample, the irradiation of the laser may cause excitation of fluorescence, resulting in either saturation of the detector or production of a poor quality Raman spectrum. In this case, it is advisable to find a different area of the sample to focus on. The edges of the dried sample are often ideal for probing due to the well-known “coffee ring effect”. This process may require trial and error for the user to understand which areas of a sample are best for analysis. Alternatively, if the Raman microscope system is advanced and allows for an automatic protocol to be set up, this is highly advisable to remove the operator-dependent data collection requirements. While a fluorescence background signal is not detrimental to the data collection procedure, ensuring the Raman spectral information is still present is ideal. In either case, maintaining a procedure which results in a consistent background signal across samples is vital.*
10. Set the parameters for collecting each spectrum within the map as follows: 1 accumulation, 30 s exposure time, at 50% of the overall laser power. The spectral range for recording spectra should be 400–1800 cm^−1^ (Figure 3). Allow the instrument to collect the entire spectral map before saving the data as a “.SPC” file. Consider also saving the spectral map as a “.WXD” file to open the file in the WiRE software if needed later on. Other file types can be chosen, as well.
11. Repeat steps 2–10 for each serum sample in the total dataset. Save all data in individual files, labeled according to which donor was analyzed, with other identifiable information included as desired.
12. For analyzing the collected spectral data, the user must have both MATLAB (MathWorks) and PLS_Toolbox (Eigenvector Research Inc., Mason, WA, USA) software programs purchased and installed on their computer. Open MATLAB and enter the code “browse” in the “Command” window. This will open PLS_Toolbox.
13. Import all spectral maps into the workspace individually using the “.SPC” files saved from each donor.
14. In the PLS_Toolbox environment, combine all data files from the calibration group into one file by selecting all files to be combined, right clicking with the mouse, and selecting “combine”. Consider renaming the combined data file.
15. Remove any spectra which display cosmic rays. To do this, double click on the combined data file, and select the “plot” Table In the “Plot Controls” window, choose “data” to display on the y-axis. Then, under the “plot” tab in the “Plot Controls” window, select “rows”. This will allow the user to scroll through each spectrum in the data file and visually check if there are any spectra that exhibit cosmic rays (Figure 4). If a cosmic ray is present, the spectrum should be removed by right clicking on that spectrum and selecting “exclude plotted”. If necessary, based on the number of spectra removed, prepare the sample again and collect an entire new spectral map, replacing the old spectral map. It was found in this study to be ideal to have as close to 50 good quality spectra for each donor as possible.
16. Label all rows (each row corresponds to an individual spectrum) from each donor according to their class–healthy or diseased. This is accomplished by double-clicking on the combined file and selecting the “row labels” Table Each row can be labeled individually, or multiple rows can be selected by right-clicking on the “class” column tab, selecting “bulk selection change,” and then selecting the rows to be labeled. Once the rows are selected, the label is changed by selecting the drop-down arrow of one row, selecting “new class” and then typing in the label. Ensure the naming of rows remains consistent.
17. In the PLS_Toolbox environment, combine all data files from the validation group into one file. Again, consider renaming the file, check for cosmic ray contamination, and label all rows from each donor according to its donor ID (in a similar manner as was performed in steps 15 and 16). Do not label the data with its class. Remove all files except for the calibration group and validation group combined files from the workspace; save the workspace.
18. In the PLS_Toolbox environment, open both the calibration dataset file and the validation dataset file. Select the “data” tab and check the boundaries of each set of spectra. That is, ensure that all recorded spectra begin and end on the same wavenumber. If there are differences, exclude the columns corresponding to wavenumbers that are not in all spectra until every spectrum has recorded intensities for the same set of wavenumbers. Columns are excluded by right clicking on the column and selecting “exclude selected”.
19. Open both combined files in Analysis by selecting “open in” under the “file” tab and then choosing “analysis.”
20. Perform preprocessing steps to each file by selecting the “preprocess” tab in the Analysis window. Then, select “x-block” followed by “custom.” Here, the user can decide which preprocessing steps to perform. Suggested preprocessing steps include: baseline correction via automatic weighted least squares (order = 4); normalization by total area; and mean centering. Alternative preprocessing steps can be used, if desired. However, it is highly recommended that mean centering is performed before chemometric analysis. Save the preprocessed data files.
  *Note that the order of implementing preprocessing steps does affect the resultant spectrum. The steps are listed in the proper order for this protocol. Additionally, if one desires to generate images of their spectra, such as those required for presentations and publications, it is best to do so before applying mean centering to the dataset (Figure 5). Lastly, the type of normalization applied, in addition to other preprocessing steps, should remain consistent across all samples. The method of normalization can be customized according to user preference.*
21. Begin the chemometric modeling process. The most basic supervised algorithm available in PLS_Toolbox is Partial Least Squares Discriminant Analysis (PLS-DA). Run the model building feature on the data file from the calibration group by selecting the “analysis” tab in the Analysis window. Under “classification” choose the “PLS-DA” model option. The cross-validation method for the model can be changed by selecting the “cross-validation” box on the right-hand side of the Analysis window. In this work, the Venetian Blind approach is chosen. The optimum number of latent variables (LVs) is predetermined by the algorithm. However, the user can change the number of LVs used to build the model, if desired. It is important to keep in mind the possibility of overfitting the model.
22. The results of cross-validation are commonly evaluated through a variety of tables. To display the results, select the icon which looks like a table in the Analysis window and says “Show confusion matrix and table”. This will depict the overall results of cross-validation (Table 1), as well as a confusion matrix which describes the number of correctly predicted and incorrectly predicted spectra for each class (not shown). The results shown in Table 1 can be converted to sensitivity, specificity, and accuracy values utilizing known calculations. Herein, the sensitivity is defined as the percentage of *mdx* spectra correctly predicted as belonging to the *mdx* class, and the specificity is defined as the percentage of control spectra correctly predicted as not belonging to the *mdx* class. Results can also be observed pictorially, by selecting the icon that resembles an Erlenmeyer flask in the Analysis window and which says “Plot scores and sample statistics”.
  *As an alternative cross-validation approach, which is typically more well-accepted and reputable, the user can decide to do a manual “leave-one-donor-out” cross-validation scheme. This will involve building the model with all donors except for one donor, which can be done by splitting the dataset manually into the calibration dataset (all donors except the one) and the validation dataset (the one left out donor). To do this, in the Analysis window under the “edit” tab, select “calibration” followed by “split into calibration/validation”. The prediction/confusion matrix results of the single left out donor is then recorded. The left out donor is then re-entered into the calibration dataset, and a new donor is selected to be the validation dataset. This process must be repeated until every donor of the calibration set has been left out. An overall confusion matrix can then be made by combining the results of all predictions. This is typically a lengthier process but is sometimes preferred, especially for smaller datasets where external validation cannot be performed.*
23. Determine if the results of cross-validation are acceptable. This is typically done by comparing the specificity, sensitivity, and accuracy results obtained to similar studies which have been reported in the literature, as well as by comparing the specificity, sensitivity, and accuracy results obtained to those which are achieved using current methods for diagnosing or identifying the disease under study.
24. If the results of cross-validation are acceptable, external validation is performed. Load the spectral data file of the validation group into the Validation X-block in the Analysis window. Run the model again, using the same parameters (number of LVs) which were determined during cross-validation. Record the results of external validation, again by regarding the confusion matrix results (Table 2).
  *If the results of external validation are poor, the model may be overfit to the data that was used to build it. At this point, the user has three options: (1) consider utilizing a different supervised classification method available in the software to build the model, (2) alter the number of LVs and cross-validation method used, or (3) expand the number of donors in the dataset. Ideally, one of these will improve the performance of the classification model.*
25. Convert the individual spectral predictions for external validation to overall donor level predictions by building a receiver operating characteristic (ROC) curve (Figure 6) using the easyROC web-tool ver. 1.3.1 available from: http://www.biosoft.hacettepe.edu.tr/easyROC/ (accessed on 06 April 2020). A ROC curve evaluates the performance of a binary classifier and is generated by plotting true positive rate values (sensitivity) against false positive rates values (1-specificty) of various decision thresholds. The most optimum threshold for discrimination would be a threshold at the position (0.00, 1.00), which corresponds to zero false positive predictions and 100% true positive predictions.
26. First, the calibration dataset predictions are exported by selecting the “file” tab in the Analysis window, and then selecting “export predictions” and finally, “calibration”.
27. Create a new Excel file with five columns. The columns will be: Donor ID number/name, Known class for each donor, Total spectra collected from each donor, Number of spectra predicted as belonging to class 1, and Percentage of spectra predicted as belonging to class 1. Fill in the table.
28. To determine the number of spectra predicted as belonging to class 1, open the calibration dataset predictions Excel file. Compare the values for “Y CV Predicted Group 1” to the threshold that was generated by the PLS-DA model. If the value for the Y CV Predicted Group 1 is equal to or greater than the threshold, that spectrum belongs to group (or class) 1. If the value is below the threshold, that spectrum belongs to group (or class) 2. Fill in the rest of the table in the new Excel file and sort the rows from smallest to largest percentage of spectra predicted as belonging to class 1.
29. Copy the column with the known class for each donor and the percentage of spectra which were predicted as belonging to class 1 into a Notepad file. Ensure there is a comma between the two values of each row, and no space.
30. Open the easyROC web-tool ver. 1.3.1 application website. Upload the Notepad file and select the delimiter to “comma.” Select the markers to reflect the percentage value. The ROC curve should now be visible.
31. The optimum ROC curve threshold is determined by calculating which point in the curve is closest in distance to the point (0.0, 1.0), and which also maximizes the number of correct predictions.
32. Apply the ROC curve threshold to the spectral predictions made for each donor of the external validation dataset. Determine the overall donor-level diagnosis (Figure 7). The threshold is used to convert individual spectral predictions for a single donor to an overall diagnosis for that donor. Specifically, if the total percentage of spectra predicted as belonging to class 1 from a single donor is equal to or greater than the threshold, than the donor itself is predicted as belonging to class 1. In the example in Figure 7, donors 1 and 2 represent blood serum samples obtained from *mdx* model mice which are 3- and 12-months old, respectively. Donors 3 and 4 represent blood serum samples which were obtained from wild-type control mice which were 3- and 12-months old, respectively. The threshold determined using the ROC curve was here found to be 77%.
33. Compare the donor-level diagnosis to the true clinical diagnosis for the donors of the external validation dataset. Then, calculate the sensitivity, specificity, and accuracy of external validation predictions at the donor level.

## 4. Expected Results

**Figure 8:**
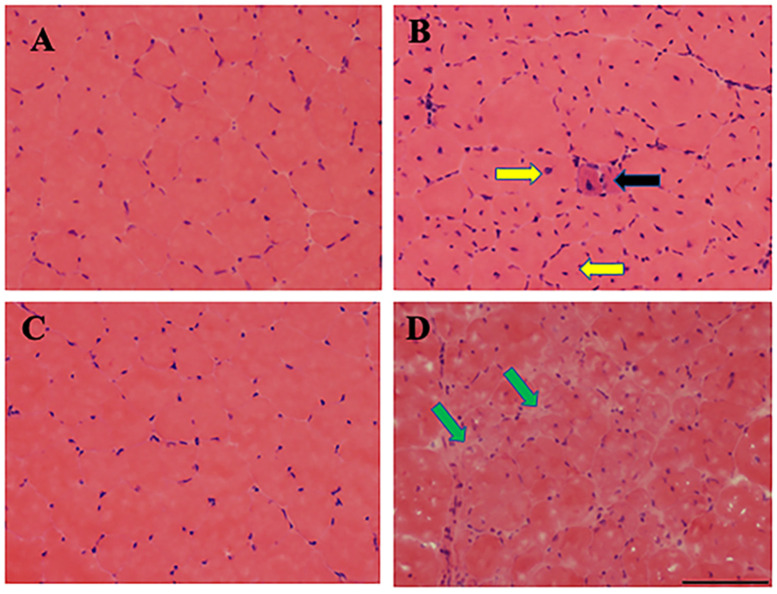
Skeletal muscle degenerates in *mdx* mouse model of DMD. Hematoxylin and Eosin (H&E) staining of Tibialis Anterior (TA) muscle sections from 3- and 12-month-old control (**A**,**C**) and *mdx* (**B**,**D**) mice are shown here. The 3-month old control muscle section shows normal fiber morphology including circular shape and absent central nuclei (**A**), whereas 3-month old *mdx* mice show muscle degeneration denoted by muscle fibers with central nuclei with smaller diameter (yellow arrows), atrophied muscle (black arrow), and more prevalent nuclei of inflammatory cells (**B**). Muscle degeneration is much more dramatic in older *mdx* muscle, as evident by the absence of normal muscle fiber structure and presence of fatty and necrotic tissues (green arrows). Scale bar: 100 μM. Reprinted with permission from N. M. Ralbovsky, P. Dey, A. Galfano, B. K. Dey, and I. K. Lednev, Diagnosis of a model of Duchenne muscular dystrophy in blood serum of *mdx* mice using Raman hyperspectroscopy, Scientific Reports, 10, 11734, Copyright (2020) Springer Nature.

The expected results from the first section of the protocol are simple: one obtains blood serum from the animal donors if followed correctly. Serum is the liquid fraction of blood, isolated after the blood sample is allowed to clot. The clotted blood is centrifuged and the resulting supernatant at the top fraction, known as serum, is collected for analysis. In this protocol, blood serum is isolated from *mdx* (C57BL/10ScSn-Dmd<*mdx*>/J) mouse model of Duchenne muscular dystrophy (DMD) and the counterpart wild type (C57BL/10ScSnJ) control mouse and analyzed using Raman spectroscopy with chemometric analysis. The 3- and 12-months old male *mdx* and male control mice are considered to be equivalent to early and advanced, respectively, DMD phenotypes in patients [31]. Although *mdx* and control mice are genotyped when purchased, it is useful to perform Hematoxylin and eosin (H&E) staining [32,33] to determine the skeletal muscle morphology (Figure 8) of the *mdx* and control mouse lines before isolation of blood serum; further, H&E staining provides additional confirmation of the health of each mouse which is necessary for building a reliable and accurate classification model.

Since we need only 5–10 µL (15–30 µL for triplicates) of serum from each animal for the Raman spectroscopy, the blood can be collected either from the Lateral Saphenous Vein or the tail vein with or without anesthesia as described [29]. The advantage of collecting blood samples using these procedures is that the same group of mice can be gradually aged and the blood can be collected at different time intervals. Blood samples can also be collected by cardiac puncture as described [29]. This cardiac puncture technique, also called intracardiac blood collection, can be suitable for the euthanized mice in which muscle samples are simultaneously harvested for analysis. The blood samples are collected in Eppendorf tubes, allowed to clot at room temperature, centrifuged, collected in a fresh tube, and stored at −80 °C until analysis. Mice are purchased from The Jackson Laboratory and housed at the animal facility, and blood serum is isolated from these animals following the standard operating procedures approved by our Institutional Animal Care and Use Committee (IACUC) and the Laboratory Animal Resources (LAR).

After the blood serum samples are isolated, the spectroscopic and chemometric analysis can begin. Raman spectroscopy is well established for providing useful information which describes the composition of a sample [34,35]. Here, Raman spectroscopy is used to investigate the entire molecular composition of the serum samples as a whole; this allows for the entire composition to be monitored rather than narrowing the focus to one or two biomolecular components. Despite the inherent specificity of Raman spectroscopy, the observed differences between Raman spectra of the same type of biological sample but obtained from healthy and diseased individuals may not be significant enough for observation [20,23]. If this situation arises, chemometrics can be used to further evaluate the Raman spectra and identify characteristics which are invisible to the human eye but which can be capitalized upon for classification efforts.

From the second portion of the protocol, the expected result is a classification model that can successfully differentiate between spectral data of blood serum obtained from healthy controls versus donors with the disease. Specifically, high quality Raman spectra, similar to those depicted in Figure 5, should be obtained. The classification model built using the Raman spectral data should undergo both cross-validation and external validation. The results should ideally indicate high levels of sensitivity, specificity, and accuracy for correctly predicting the class of individual Raman spectra, as well as overall donors. Troubleshooting suggestions are posited throughout the protocol. Further information and resources for using both PLS_Toolbox and MATLAB can be found on each company’s respective website. Once the algorithm is built, spectral data of blood serum collected from new, unknown, donors may be loaded into the model for accurate diagnostic predictions. Thus, the overall expected result is the development of an accurate and objective method using Raman spectroscopy in combination with chemometrics which can be used to decide whether or not an individual has Duchenne muscular dystrophy. This protocol provides the first step toward pursing the investigation of DMD within human patients; future studies will focus on applying the method of Raman spectroscopy with chemometric analysis for identifying DMD within serum samples obtained from human patients in a non-invasive, accurate, and objective manner.

## Figures and Tables

**Table 1:** Example of the results of cross-validation. TPR: true positive rate; FPR: false positive rate; TNR: true negative rate; FNR: false negative rate.

Class	TPR	FPR	TNR	FNR
**Control**	0.946	0.0483	0.952	0.0536
***mdx***	0.952	0.0536	0.946	0.0483

**Table 2:** Example of the results of external validation. TPR: true positive rate; FPR: false positive rate; TNR: true negative rate; FNR: false negative rate.

Class	TPR	FPR	TNR	FNR
**Control**	0.870	0.000	1.00	0.130
***mdx***	1.00	0.130	0.870	0.000

## Data Availability

Not applicable.
